# Inferring Bladder Cancer Evolution from Mucosal field Effects by Whole-Organ Spatial Mutational, Proteomic, and Metabolomic Mapping

**DOI:** 10.21203/rs.3.rs-3994376/v1

**Published:** 2024-04-10

**Authors:** Bogdan Czerniak, Sangkyou Lee, Sung Yun Jung, Pawel Kus, Jolanta Bondaruk, June Lee, Roman Jaksik, Nagireddy Putluri, Khanh Dinh, David Cogdell, Huiqin Chen, Yishan Wang, Jiansong Chen, Neema Nevai, Colin Dinney, Cathy Mendelsohn, David McConkey, Richard Behringer, Charles Guo, Peng Wei, Marek Kimmel

**Affiliations:** The University of Texas MD Anderson Cancer Center; The University of Texas MD Anderson Cancer Center; Baylor College of Medicine; Silesian Univerity of Technology; The University of Texas MD Anderson Cancer Center; The University of Texas MD Anderson Cancer Center; Silesian University of Technology; Baylor College of Medicine; Irving Institute for Cancer Dynamics, Columbia University; The University of Texas MD Anderson Cancer Center; The University of Texas MD Anderson Cancer Center; The University of Texas MD Anderson Cancer Center; The University of Texas MD Anderson Cancer Center; The University of Texas MD Anderson Cancer Center; The University of Texas MD Anderson Cancer Center; Columbia University; Johns Hopkins Greenberg Bladder Cancer Institute, Johns Hopkins University; University of Texas M. D. Anderson Cancer Center; The University of Texas MD Anderson Cancer Center; The University of Texas MD Anderson Cancer Center; Rice University

## Abstract

Multi-platform mutational, proteomic, and metabolomic spatial mapping was used on the whole-organ scale to identify the molecular evolution of bladder cancer from mucosal field effects. We identified complex proteomic and metabolomic dysregulations in microscopically normal areas of bladder mucosa adjacent to dysplasia and carcinoma *in situ*. The mutational landscape developed in a background of complex defects of protein homeostasis which included dysregulated nucleocytoplasmic transport, splicesome, ribosome biogenesis, and peroxisome. These changes were combined with altered urothelial differentiation which involved lipid metabolism and protein degradations controlled by PPAR. The complex alterations of proteome were accompanied by dysregulation of gluco-lipid energy-related metabolism. The analysis of mutational landscape identified three types of mutations based on their geographic distribution and variant allele frequencies. The most common were low frequency α mutations restricted to individual mucosal samples. The two other groups of mutations were associated with clonal expansion. The first of this group referred to as β mutations occurred at low frequencies across the mucosa. The second of this group called γ mutations increased in frequency with disease progression. Modeling of the mutations revealed that carcinogenesis may span nearly 30 years and can be divided into dormant and progressive phases. The α mutations developed gradually in the dormant phase. The progressive phase lasted approximately five years and was signified by the advent of β mutations, but it was driven by γ mutations which developed during the last 2–3 years of disease progression to invasive cancer. Our study indicates that the understanding of complex alterations involving mucosal microenvironment initiating bladder carcinogenesis can be inferred from the multi-platform whole-organ mapping.

## INTRODUCTION

The molecular mechanisms that initiate carcinogenesis involve microscopically normal appearing tissue and are collectively referred to as field effects.^1,2^ Their characterization may facilitate the development of early detection, prevention, and treatment strategies intercepting carcinogenesis in its early phases before it progresses to clinically aggressive and often uncurable disease. Understanding of these initiating events is not possible unless they are analyzed in the geographic spatial frame of mucosal changes in the entire organ. Bladder cancer is a particularly useful model for such studies as it develops in the epithelial lining, the urothelium, of the anatomically simple organ facilitating combined geographic microscopic and multi-platform genomic mapping. We and others have recently characterized this field cancerization using genomics.^3–7^ These studies revealed that regionally restricted clonal expansions are present in areas of the urothelium that appear phenotypically normal, and that these changes are associated with mutations in cancer driver genes, particularly those that regulate chromatin structure.^3,4,8^ These changes are associated with alterations in RNA expression that appear to disrupt innate immunity among other pathways and cause T-cell exhaustion.^3,8^ However, very little is known about how these genomic changes alter urothelial biology, which requires a deeper understanding of the downstream effects on protein expression and metabolism. Here we provide the first descriptions of the proteomic and metabolomics profiles of bladder cancer evolution from mucosal field effects in the context of their mutational landscape on the whole-organ scale.

## RESULTS

### Preparation of Whole-Organ Cystectomy for Spatial Mapping

**Figure 1:**
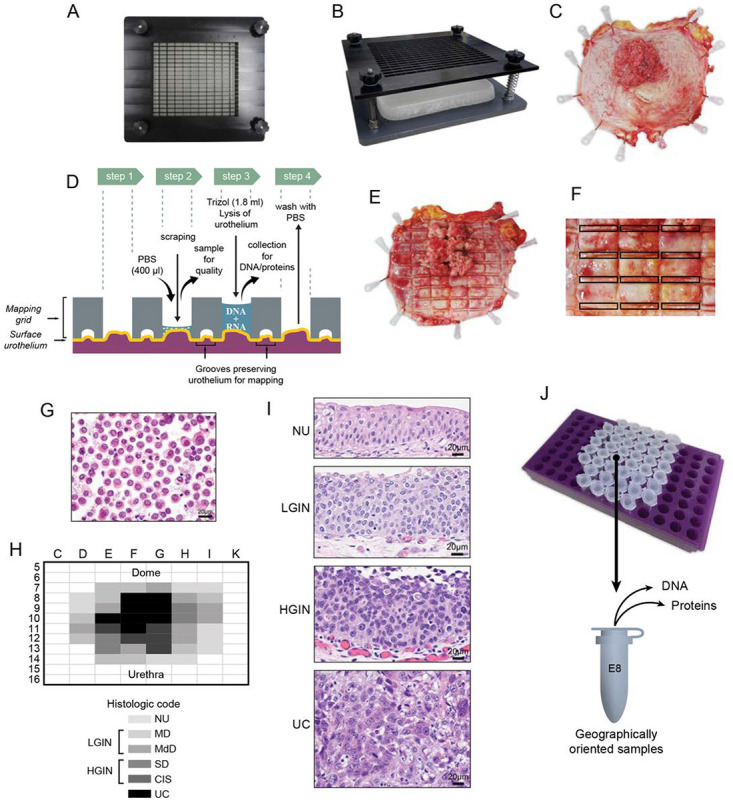
Preparation of whole-organ maps for multi-platform genomic, proteomic, and metabolomic profiling. **(a)** Top view of the mapping grid for whole-organ sampling. **(b)** Oblique view of the mapping grid. **(c)** Photograph of an open cystectomy specimen pinned down to a paraffin block showing a fungating tumor involving the posterior bladder wall. **(d)** Diagram showing the details of the mapping grid preserving the urothelium for histologic mapping and permitting simultaneous DNA/RNA and protein extraction. **(e)** Open cystectomy specimens shown in C with the impressions of the mapping grid for histologic sampling. **(f)** Enlarge mucosal area with the impression of the mapping grid showing the sampling pattern for histologic mapping of the mucosa. **(g)** Urothelial single cell suspension after sample collections from geographically mapped mucosal areas used for DNA/RNA and protein extractions. **(h)** A whole-organ histologic map prepared by sampling of the entire bladder mucosa of the cystectomy specimen shown in **C**. **(i)** Representative microscopic images corresponding to NU, LGIN, HGIN, and UC. **(j)** Geographically mapped urothelial cell suspensions corresponding to histologic map shown in **H** used for DNA/RNA and protein extractions.

To molecularly characterize the evolution of bladder cancer to mucosal field effect on the whole-organ scale, we collected geographically annotated mucosal samples from a representative cystectomy specimen with invasive bladder cancer (Fig. 1A–J). For whole-organ mapping, the resected human bladder was open along the anterior wall and pinned down to a paraffin block. Then a mapping grid was applied which separated the mucosal areas into 1×2 cm wells allowing DNA and protein to be extracted while simultaneously preserving the urothelium for microscopic inspection from which the histologic map of the entire bladder mucosa and invasive cancer was reconstructed. The samples were microscopically classified as normal urothelium (NU), *in situ* preneoplastic conditions referred to as low and high-grade intraurothelial neoplasia (LGIN and HGIN) respectively, and invasive urothelial carcinoma (UC). Geographically oriented samples were analyzed by bulk whole-exome DNA sequencing and proteomic sequencing. Germline DNA from peripheral blood samples was used as a reference for DNA sequencing. Microscopically normal urothelium harvested from ureters of nephrectomy specimens from patients without urothelial neoplasia was used as reference from proteomic sequencing.

### Mutational Landscape of field Effects and their Evolution to Carcinoma

**Figure 2:**
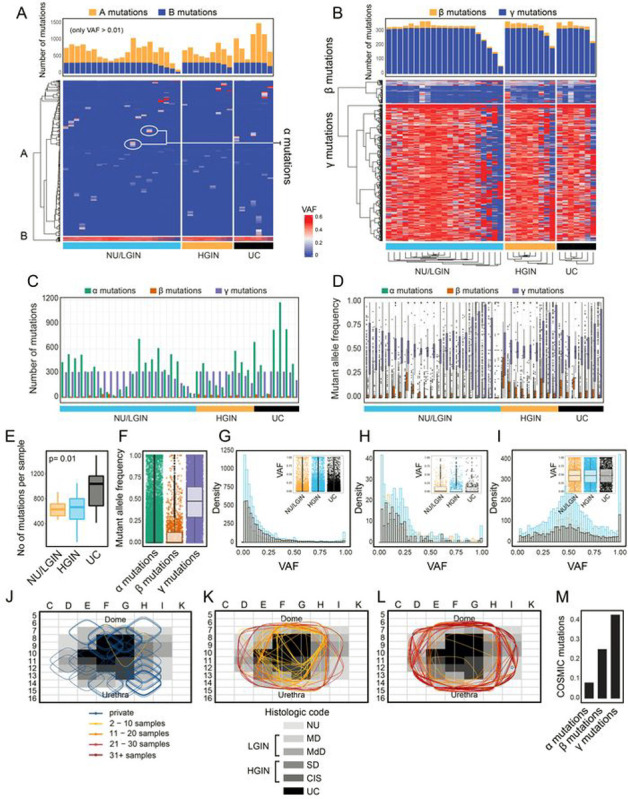
Mutational landscape of bladder cancer evolution from field effects. **(a)** Heat map of non-silent mutations showing VAFs in individual mucosal samples. Number of mutations in individual mucosal samples are shown in the top diagram. **(b)** Heat map of VAFs ≥0.01 in genes showing variant alleles in at least three mucosal samples. Number of β and γ mutations in individual mucosal samples are shown in the top diagram. **(c)** Number of α, β, and γ mutations in individual mucosal samples. **(d)** VAFs of α, β, and γ mutations. **(e)** Box plot analysis of number of mutations in mucosal samples classified as NU/LGIN, HGIN, and UC. **(f)** VAFs of α, β, and γ mutations. **(g)** Histogram showing the clonality of VAFs in α mutation. Inset, box plot of VAFs of α mutations in three groups of samples corresponding to NU/LGIN, HGIN, and UC. **(h)** Histogram showing the clonality of VAFs in β mutations. Inset, box plot of VAFs of β mutations in three groups of samples corresponding to NU/LGIN, HGIN, and UC. **(i)** Histogram showing the clonality of VAFs in γ mutations. Inset, box plot of VAFs of γ mutations in three groups of samples corresponding to NU/LGIN, HGIN, and UC. **(j)** Spatial distribution of 50 randomly selected α mutations superimposed on the histologic map of the cystectomy. **(k)** Spatial distribution of β mutations superimposed on the histologic map of the cystectomy. **(l)** Spatial distribution of 50 randomly selected mutations superimposed on the histologic map of the cystectomy. For **g**, **h**, and **l**; Orange, NU samples of the map; Blue, HGIN samples of the map; Black, UC samples of the map. For **j, k**, and **l**; The color-coded ovals represent mutation spread. **(m)** Fractions of COSMIC cancer mutations in **α, β,** and **γ** mutations.

Whole-exome sequencing of DNA from geographically mapped mucosal samples identified nonsynonymous variant alleles in 12,764 (12,022 SNVs, 450 inserts, and 292 deletions) genomic loci. The heat map which included their geographic distribution and variant allele frequencies (VAFs) of these mutations of individual mucosal samples is shown in Fig. 2A. Based on their VAFs and geographic distribution, we separated these mutations in to two major groups. The first group comprised of cluster A consisting of mutations with low VAFs confined to individual mucosal samples referred to as α mutations **(Table S1)**. The second group comprised of cluster B consisting of mutations expanding across the bladder mucosa. Cluster B mutations were further divided into two subgroups referred to as β and γ **(Table S2, S3;** Fig. 2B). Mutations of cluster β were expanding across the bladder mucosa but their VAFs were consistently low (< 20%) and did not increase in their frequencies with progression to HGIN and UC. On the other hand, mutations of cluster γ formed a plaque involving large areas of bladder mucosa with consistently high level (> 20%) of VAFs. Overall, based on the geographic distribution and the VAFs, three distinct types of mutations were identified and referred to as α, β, and γ **(Table S4;** Fig. 2A–D). The mutations of α type were the most frequent (12,431) comprising 11,693 SNVs, 448 insertions, and 285 deletions. There were only 54 β mutations with 51 SNVs, 1 insertion, and 2 deletions. There were 324 γ mutations with 315 SNVs, 2 insertions, and 7 deletions. Although the VAFs of γ mutations were high in all three groups of samples corresponding to NU/LGIN, HGIN, and UC there was a major increase in the number of mutations with progression to HGIN and UC (Fig. 2C–F). Most of those mutations were of γ type.

In order to address the issue of preferential selection of the mutations and their potential driver’s role we analyzed their spatial distribution and VAFs in different fields of the map. We focused on the distribution patterns of α, β, and γ mutations addressing the hypothesis that some of these mutations maybe under positive selection while others are mere hitchhikers (Fig. 2G–I). First, we additionally classified the mutations into private (present in only one field), regional (present in 2–10 or 11–20 fields), and widespread mutations (present in 21–30 or > 30 fields). The *α* mutations were almost exclusively private while the γ mutations were almost exclusively widespread. Some of the *β* mutations were regional while others were widespread.

The *α* mutations, in addition to being private have right-skewed VAF distributions typical of proliferating cells with neutral mutations (Fig. 2G, J; **Figure S1A)**.^9^ Similarly, the VAFs of β mutations were right skewed but their spread was at least regional involving several mucosal samples (Fig. 2J, K; **Figure S1B)**. The VAFs of the widespread mutations were binomially distributed in most fields, which signifies a secondary clone that acquired positive selective advantage (Fig. 2H, I; **Figure S1C)**.^9^ In some fields the distribution of VAFs for γ mutations were almost uniform which is likely the sign of widespread genome copy number dysregulation **(Figure S1C)**. The VAFs of β mutations in individual samples were in general highly irregular but in some fields have more right skewed pattern while in others have binomial distributions. This pattern suggests the mixture of clones with different proliferative advantage. Overall, there was a progressive increase of COSMIC driver mutations in mutations α, β, and γ.

### Mechanisms of Mutagenesis involved in Bladder Cancer Evolution from field Effects

**Figure 3:**
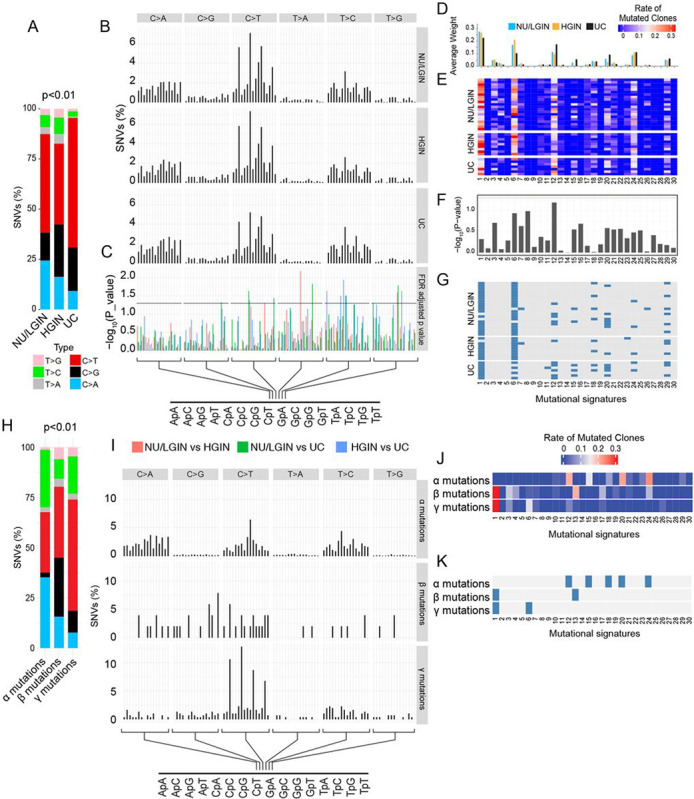
Modeling of bladder cancer mutational landscape evolution from mucosal field effects. **(a)** Bar graphs showing the distribution of all nucleotide substitutions in relation to cancer evolution from NU/LGIN through HGIN to UC. It shows an increase in the number of C>T mutations (Fisher’s exact test; p<0.01) in the progression to HGIN and UC. **(b)** Proportion of SNVs in nucleotide MOTIFs for each category of substitution in sets of samples corresponding to NU/LGIN, HGIN, and UC. **(c)** FDRs for nucleotide motifs in the progression of neoplasia from NU/LGIN through HGIN to UC. **(d)** Weight scores for mutagenesis signatures in samples corresponding to NU/LGIN, HGIN, and UC. **(e)** Weight scores for mutagenesis signatures in individual samples of bladder mucosa. **(f)** Significance of mutational signatures in the progression of neoplasia from NU/LGIN through HGIN, to UC. **(g)** Significance of contributions for mutagenesis signatures in individual samples after bootstrapping. The blue boxes indicate p value <0.05. **(h)** Bar graphs showing the distribution of nucleotide substitutions in α, β, and γ mutations. **(i)** Proportion of SNPs in nucleotide motifs for each category of substitutions for α, β, and γ mutations. **(j)** Weight scores of mutagenesis signatures for α, β, and γ mutations. **(k)** Significance of contributions of mutagenesis signature associated with α, β, and γ mutations after bootstrapping. The blue boxes indicate p value <0.05. For **A** and **H** p values were calculated using a test of proportions. For **C** and **F** p values were calculated using Wilcoxon test and Kruskal-Wallis test, respectively.

To characterize the mutagenesis signatures in the evolution of bladder cancer from field effects we first analyzed six single-based nucleotide substitutions (C > A, C > G, C > T, T > A, T > C, and T > G) and their context motifs in all mucosal samples (Fig. 3A–C). The frequency of C > T substitutions increased at the transition from NU/LGIN to HGIN and UC with significant changes in 12 mutational signatures. Signatures 1, 6, 12, 20, and 24 appeared to be the most dominant with signature 1 having the highest weight scores (Fig. 3D–F). To evaluate the contributions of individual mutagenesis pattern to the mucosal mutational landscape we performed bootstrapping and calculated the p value to assess their significance (p < 0.005 was considered statistically significant). This approach confirmed the dominance of signatures 1 and 6 (Fig. 3G). The analysis of α, β, and γ mutations identified a significant increase of C > T substitutions in γ mutations (Fig. 3H). Similarly, it was evident that these three groups of mutations were associated with distinct mutational signatures confirming their development through distinct mutational mechanisms (Fig. 3I–K).

### Modeling of Bladder Cancer Evolution from Mucosal field Effects

**Figure 4:**
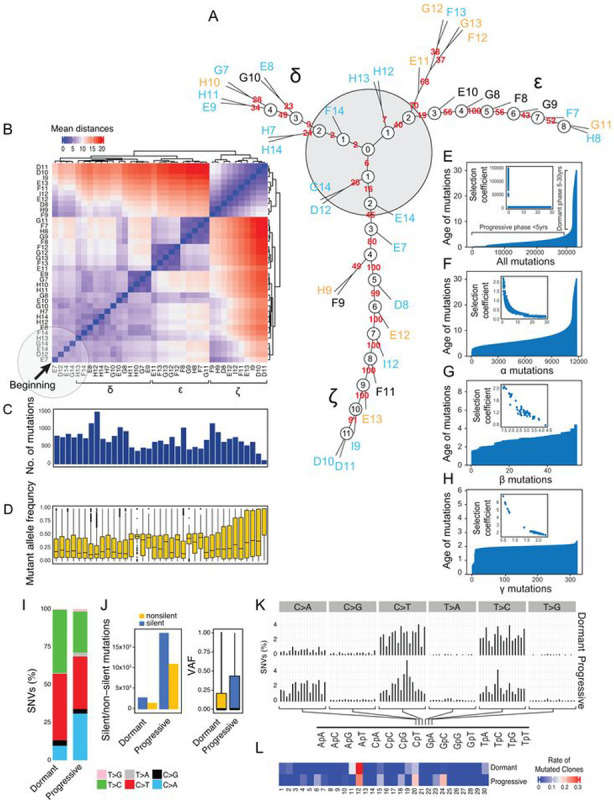
Modeling of bladder cancer evolution from its mutational landscape. **(a)** Parsimony analysis showing an evolutionary tree of expansion of successive clone of cells in the field effects corresponding to NU/LGIN along three branches designated as δ, ε, and ζ. The hypothetical beginning of the process is designated as node 0 and depicted as a gray shadow circle. **(b)** The heat map of genetic distance for successive clones with three clusters corresponding to branches δ, ε, and ζ in **a**. The beginning of the process is in the lower left corner indicated by the black arrow. **(c)** Number of mutations in individual samples of the cystectomy organized in the same order as in **b**. **(d)** VAFs in individual samples of the cystectomy organized in the same order as in **b**. Note the increase in VAFs in samples corresponding to the branch ζ. **(e)** Ages of all synonymous and non-synonymous mutations predicted by mathematical modeling. Inset, the selection coefficient in relation to the predicted mutation age. **(f)** Ages of mutations α predicated by mathematical modeling. Inset, the selection coefficient of α mutations. **(g)** Ages of mutations β predicated by mathematical modeling. Inset, the selection coefficient of β mutations. **(h)** Ages of mutations γ predicated by mathematical modeling. Inset, the selection coefficient of γ mutations. **(i) B**ar diagrams of nucleotides substitutions in dormant and progressive phases of bladder carcinogenesis. **(j)** Number of silent and nonsilent mutations in dormant and progressive phases of bladder carcinogenesis (left diagram). Vafs in dormant and progressive phases of bladder carcinogenesis (right diagram). **(k)** Proportion of SNVs in nucleotide motifs for each category of substitution in dormant and progressive phases of bladder carcinogenesis. **(l)** Weight scores for mutagenesis patterns in dormant and progressive phases of bladder carcinogenesis.

To analyze the mutational pattern of clonal evolution of bladder cancer from field effects we used all non-silent and silent mutations to construct an evolutionary tree. This revealed a complex branching pattern corresponding to multiple waves of clonal expansion with 4–11 nodes evolving along three distinct branches referred to as δ, ε, and ζ (Fig. 4A). The process evolved from the hypothetical node 0 in the center and neighboring nodes 1 and 2 of the three branches representing incipient events of carcinogenesis. The heat map of genetic distance among individual samples showed the gradual evolution of the mutational landscape from the initiating events of the left lower corner with three clusters of the mutational landscape corresponding to the three branches of the evolutionary tree (Fig. 4B). The branch ζ appeared to be most mutationally active showing an increase of their VAFs (Fig. 4C,D).

To answer the question of how long bladder cancer takes to develop, we applied a mathematical modeling algorithm to a whole-organ mutational landscape. We used successive waves of clonal evolutions of the parsimony trees applying a time-continuous Markov branching process.^10^ This provided time scale of cancer evolution from field effects based on maximal parsimonious principles. Initially, we performed the analysis using all synonymous and nonsynonymous mutations (Fig. 4E). These analyses showed that cancer evolved from field effects over approximately 30 years and the age-related curve of mutations had a left-skewed pattern with the mutations gradually developing over nearly three decades. Using the time scale of mutations, the process of tumor evolution could be divided into two major phases referred to as dormant and progressive. The first, older dormant phase in which mutations developed over approximately two decades, involved mutations which were characterized by low selection coefficients consistent with their marginal proliferative advantage. Second, the more recent and progressive phase was less than five years old and was characterized by a large number of mutations with high selection coefficients consistent with their clonal expansion and high proliferative advantage. We repeated the modeling analysis selectively using the three types of mutations referred to as α, β, and γ (Fig. 4F–H). These analyses have shown that the α mutations were the oldest and gradually developed over 30 years (Fig. 4F). Large proportion of them had low selection co-efficient consistent with the minimal proliferative advantage and the fact they were involving small mucosal areas. The mutations of β type emerged at the transition from dormant to progressive phase and developed over the period of less than five years (Fig. 4G). Large proportion of them showed increased selection coefficient consistent with proliferative advantage and clonal expansion. The mutation of γ type were the younger and they developed over the last two years before cancer diagnosis (Fig. 4H). These mutations were characterized by high proliferative coefficients consistent with the proliferative advantage and the involvement in the last two years of the progressive phase. Overall, these analyses have shown that dormant and progressive phases of bladder carcinogenesis were characterized by distinct involvement of α, β, and γ mutations.

To address the issue of potential distinctive involvement of different mutagenesis mechanisms in different phases of bladder carcinogenesis we performed a detailed analysis of the mutational signatures in dormant and progressive phases of the disease. These analyses showed that the progressive disease was characterized by an increased C > A substitutions combined with major increase of both silent and non-silent mutations and their VAFs (Fig. 4I, J). Dormant and progressive phases of bladder carcinogenesis showed the involvement of distinct mutagenesis signatures confirming the concept that distinct mutational mechanisms are switched on in a background of dormant field effect driving the progression to clinically aggressive bladder cancer (Fig. 4K, L).

### Proteomic Profile of Bladder Cancer Evolution from Mucosal field Effects

**Figure 5:**
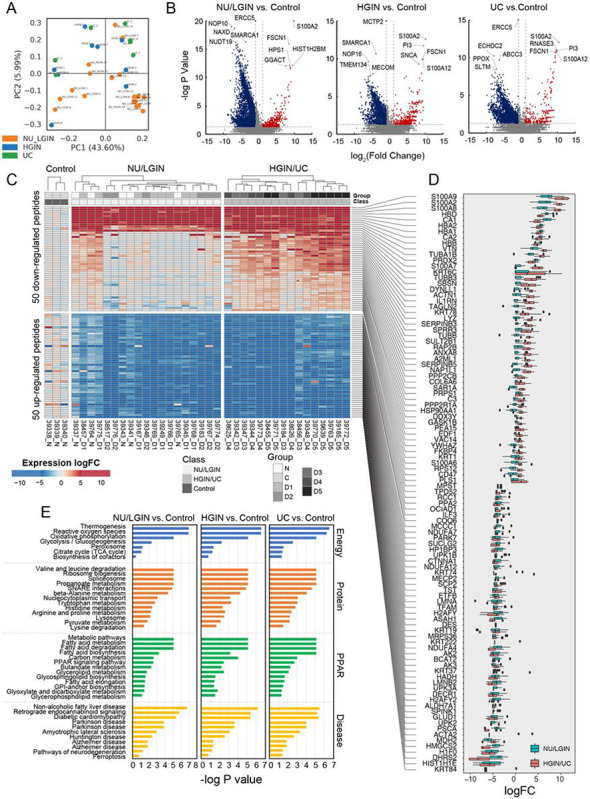
Proteomic profile of bladder cancer evolution from mucosal field effects. **(a)** PCA analysis of protein expression data for all mucosal samples. **(b)** Volcano plot of all annotated proteins comparing log2 fold change with −log p value in NU/LGIN samples versus control (left panel), HGIN versus control (middle panel), and UC versus control (right panel). **(c)** Heat map of top 50 up regulated and top 50 down regulated proteins. **(d)** Box plot analysis of top 50 up regulated and top 50 downregulated proteins shown in panel c depicting whole change of NU/LGIN sample and HGIN/UC samples compared to controls. **(e)** Monotonically dysregulated KEGG protein pathways showing −log p value for comparisons of NU/LGIN versus control (left panel), HGIN versus control (middle panel), UC versus control (right panel).

Proteomic profiling of 38 geographically mapped mucosal samples from a cystectomy specimen identified 8,475 distinctive proteins, which are listed in **Table S5**. The heatmap of their expression patterns are shown in **Figure S3A, B**. The proteome coverage for individual mucosal samples ranged from 150 to 7,123 proteins **(Figure S2A)**. The samples from mucosal areas with more than 2,500 identified proteins were selected for downstream analysis and grouped into NU/LGIN, HGIN, and UC. The protein extracted from normal ureters of nephrectomy specimens from patients without urothelial neoplasia were used as a reference. The proteome abundancy distribution was consistent across 38 selected mucosal samples as shown in **Figure S2B**. The number of gene products and Peptide Spectrum Matches (PSMs) in individual mucosal samples and the rank order of normalized proteins are shown in **Figure S2C, D**. The principal component analysis of proteome composition showed a clear separation among NU/LGIN versus HGIN and UC samples (Fig. 5A). The HGIN and UC samples co-clustered together. Gene ontology analysis of cellular components showed that we recovered approximately 50% proteins of the reported total number of proteins in each cellular compartment **(Figure S2E)**. In the analysis of proteins, we focused on the monotonic changes of protein expression which paralleled the progression of neoplasia from NU/LGIN through HGIN to UC. The total numbers of proteins with monotonic upregulation or downregulation in this progression were: 2,343, 2,413, and 2,504 respectively which were analyzed by iPathwayguide program. The top 50 monotonically upregulated and downregulated proteins are shown in Fig. 5C, D. By using this approach, we identified 42 dysregulated protein pathways in the mucosal field effects corresponding to NU/LGIN, which continued to be progressively dysregulated in the evolution of neoplasia through HGIN to UC **(Table S6)**. The dysregulated pathways converging on energy and protein homeostasis were complemented by tissue differentiation program defects (Fig. 5E). The example of downregulated energy pathways included oxidative phosphorylation, valine, leucine and isoleucine degradation, TCA cycle, and thermogenesis. The protein homeostasis defects include dysregulated nucleocytoplasmic transport, splicesome, ribosome biogenesis, and peroxisome. The signature dysregulated pathway involved in tissue differentiation, lipid metabolism and protein degradation represents PPAR signaling.

### Metabolomic Analysis of Bladder Cancer Evolution from field Effects

**Figure 6:**
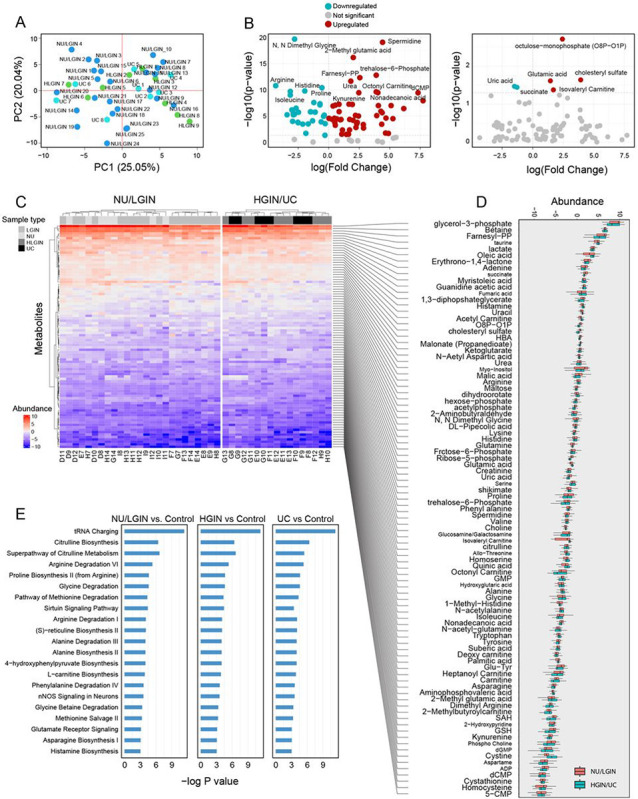
Metabolomic profile of bladder cancer evolution from mucosal field effects. **(a)** PCA analysis of metabolite abundance data for all mucosal samples. **(b)** Volcano plot of all identified metabolites comparing log2 fold change with −log p value for all mucosal samples versus control (left panel), and for HGIN/UC versus NU/LGIN. **(c)** Heat map of all identified metabolites in mucosal samples of the cystectomy. **(d)** Box plot analysis showing fold change of metabolites abundance in NU/LGIN and HGIN/UC compared with control. **(e)** Metabolomic KEGG pathways monotonically dysregulated in evolution of bladder cancer from field effects showing −log p value for NU/LGIN versus control (left panel), HGIN versus control (middle panel), and UC versus control (right panel).

Targeted metabolomic profiling of 38 geographically mapped mucosal samples from a cystectomy specimen using positive and negative ionization modes (Electrospray Ionization, ESI) identified 91 metabolites **(Table S7)**. Subsequently, the peak areas of metabolites were normalized using internal standards for further analysis. The Principal Component Analysis (PCA) showed the clustering distinction among the samples from NU/LGIN, HGIN, and UC groups and revealed a preferential clustering of HGIN and UC samples (Fig. 6A). Differentially expressed metabolites visualized on the PCA plot, represented their log fold change values, with significantly upregulated and downregulated metabolites highlighted. The differentially expressed metabolites of the mucosal samples are shown in the volcano plots as compared to control samples and for comparisons between HGIN/UC versus NU/LGIN in Fig. 6B. The total number of measured metabolites and their expression patterns in individual mucosal samples classified as NU/LGIN and HGIN/UC are shown in a heatmap and box plot in Fig. 6C,D. Among the top five monotonically upregulated metabolites were glycerol-3-phosphate, betaine, farnesyl-PP, taurine, and lactate. Among the top five monotonically downregulated proteins were 5-CMP, homocysteine, cystathionine, dCMP, and ADP. Furthermore, we conducted pathway analysis for three comparisons: 1) NU/LGIN vs. control, 2) HGIN vs. control, and 3) UC vs. control (Fig. 6E). The top five monotonically enriched pathways were tRNA charging, citrulline biosynthesis, superpathway of citrulline metabolism, arginine degradation VI, and proline biosynthesis II (from arginine). The analysis of enrichment scores in individual mucosal samples for metabolites using single sample gene set enrichment analysis (ssGSEA) identified 74 monotonically dysregulated pathways **(Table S8; Figure S4)**. Among the top five activated pathways were glycerolipid metabolism, steroid biosynthesis, terpenoid backbone biosynthesis, taurine and hypotaurine metabolism, and cAMP signaling. Among the top five downregulated pathways were platelet activation, cysteine and methionine metabolism, fatty acid elongation, fatty acid biosynthesis, and fatty acid degradation. Interestingly, elevation of lactate and activation of glycolysis was already evident in microscopically normal appearing areas of bladder mucosa implicating the emergence of Warburg phenotype in the field effects. These analyses taken together indicate that there were complex alterations of gluco-lipid energy-related metabolism in field effects dysregulating mucosal microenvironment in the incipient phases of bladder carcinogenesis.

## DISCUSSION

This study provided a proof of principle for the validity and feasibility of multi-platform mutational, proteomic, and metabolomic analysis on the whole-organ scale to infer the molecular profile of bladder cancer development from mucosal field effects. It provides evidence for time modeling of mutational landscape in the context of complex alterations involving protein homeostasis and tissue differentiation program combined with dysregulation of gluco-lipid energy-related metabolism. Proteomic profiling of bladder cancer tissue performed by others revealed subtype and stage-related differences converging on glucolipid metabolism.^11–13^ Whole-organ spatial geographic analyses revealed that bladder carcinogenesis may develop innocuously over several decades and can be divided into dormant and progressive phases. The mutational landscape develops gradually via multiple waves corresponding to distinct successive clones of cells which may develop along several distinct branches. The most common are low frequency α mutations restricted to individual mucosal samples. It is very likely they represent the progeny of individual uro-progenitor cells. The α mutations are the oldest and gradually develop over three decades. The other two groups referred to as β and γ mutations were associated with intramucosal clonal expansion. The first of these two groups were β mutations with low frequency of VAFs typically comprising of < 10% of cells. The emergence of clone with β mutations was associated with the transition to the progressive phase during the last five years of carcinogenesis. The second group referred to as γ mutations appeared to be drivers of the progressive phase emerging 2–3 years before the progression to invasive cancer.

Highly variable mutagenesis signatures were identified in individual mucosal samples but signatures 1 and 6 were dominant.^14^ Moreover, the development of α, β, and ϒ mutations were driven by distinct mutational mechanisms. Similarly, the transition from dormant to progressive phase of carcinogenesis was associated with the involvement of different mutagenesis signatures confirming that the progressive phase of bladder carcinogenesis developed through the activation of distinct mutational mechanisms.

These mutational landscape changes developed in a background of complex proteomic and metabolomic dysregulations. Overall the proteome alterations involved defects of protein homeostasis including dysregulated nucleocytoplasmic transport, splicesome, ribosome biogenesis, and peroxisome. Interestingly these changes were associated with dysregulated urothelial differentiation controlled by PPAR and involved lipid metabolism and protein degradations.^15,16^ The proteome alterations were accompanied by equally complex dysregulation of glycerolipid metabolism, steroid biosynthesis, terpenoid backbone biosynthesis, taurine and hypotaurine metabolism, and cAMP signaling. The top dysregulated metabolomic pathways involved cysteine and methionine metabolism, fatty acid elongation, fatty acid biosynthesis, and fatty acid degradation including activated glycolysis signifying complex alterations of gluco-lipid energy-related metabolism in field effects complementing the dysregulated mucosal microenvironment in the incipient phases of bladder carcinogenesis. These observations are in synchrony with the work by others indicating that early events of carcinogenesis involve complex metabolic alterations converging on glycolysis and oxidative phosphorylation favoring early clonal expansion of cells with the Warburg phenotype.^17–20^ Interestingly, recent lipidomic profiling studies identified unique lipid signatures of bladder cancer associated with a clinical stage and ethnicity.^21–23^

## MATERIAL AND METHODS

### Experimental Model and Subject Details

Human samples and clinical data were collected according to the laboratory protocols approved by the Institutional Review Board of The University of Texas MD Anderson Cancer Center. The whole-organ histologic, genomic, and proteomic mapping (WOHGPM) was performed on the radical cystectomy specimen from 92-year-old white male patient with high-grade urothelial carcinoma invasive into muscularis propia (T_3_) as previously described. In brief, the preparation of cystectomy specimen for whole-organ histologic mapping combined with DNA/RNA and protein extraction followed the steps illustrated in **Supplementary Data** Fig. 1. The cystectomy specimen was open longitudinally along the anterior wall of the bladder and pinned down to a paraffin block. Then the mapping grid was superimposed and pressed down over the bladder mucosa with mechanical screws. The mapping grid provided sealed wells that separated mucosal areas into 1×2cm (2cm^2^) rectangles. Phosphate-buffered saline (PBS) with 0.25% Trypsin (1ml) was poured into each well and the surface urothelium was scraped. The fluid was collected into Eppendorf tubes and incubated at 37°C for 30 minutes. The urothelial cell clusters and tissue fragments were removed by passing the cell suspension through the 70μm cell strainer. Subsequently, the red blood cells were removed by Ficoll gradient centrifugation. The single cell suspensions were washed twice in PBS containing 0.04% BSA and resuspended in 600μl of PBS. A small proportion of this fluid was used for cytospin preparation to assess the purity and quality of cell suspension. In areas of mucosa which contain grossly recognizable tumor, the tumor tissue was collected by direct dissection from the bladder wall, cut into small pieces and processed as described above. The final 600μl cell suspensions were divided into three parts and kept frozen until processed for DNA, protein, and metabolite extractions. For DNA extraction the cell suspensions were defrosted and treated with triazol reagents. The mapping grid was removed from the surface of the bladder which was then fixed in formalin overnight. The grooves at the bottom of the mapping grid left permanent impression on the bladder surface and preserved the urothelium for microscopic inspection and histologic mapping of the entire bladder mucosa. Paraffin embedded sections corresponding to mapping grooves were collected and were stained with hematoxylin & eosin to evaluate the distribution of microscopically normal urothelium *in situ* precursor lesions and urothelial carcinoma. The intraurothelial lesions were dichotomized into low- and high-grade categories referred to as low-grade intraurothelial neoplasia (LGIN) and high-grade intraurothelial neoplasia (HGIN) as previously described.^24,25^ Samples with tumor tissue were classified according to the two-tier histologic grading system of the World Health Organization (WHO) referred to as low- and high-grade.^26^ The growth pattern of papillary versus solid and the depth of invasion were recorded. Levels of invasion were defined according to the Tumor Node Metastasis (TNM) Staging System.^27,28^

There were two steps of quality check controls which comprised of the overall assessment of the cystectomy specimens and the quality of final DNA/RNA and protein preparations for genomic and proteomic profiling. In the first step the cystectomy specimen was assessed in terms of the representation of the whole spectrum of the *in situ* precursor lesions and tumor samples as well as purity of urothelial and tumor cell preparations. In the second step the quality of DNA/RNA preparations were verified using NanoDrop, Bioanalyzer, and Qubit.

### Whole-Exome Sequencing and Data Analysis

Whole-exome sequencing as well as downstream data analysis were performed as outlined in recent cancer sequencing project publications. In brief, whole-exome sequencing was performed by using an Illumina NovaSeq6000 sequencer using high output flow cell with an average coverage across the samples of 300X ± 85.3 SD. The initial alignments of reads to GRCh38 reference genome was performed with BWA-MEM (version 0.7.12). The Genome Analysis Toolkit (GATK, version 3.4–46) was used to generate realigned and recalibrated BAM files. MuTect2 and Oncotator (version 1.8.0.0) were used to identify mutations.

### Mutational Signatures

The analyses of mutational signatures were performed as previously described.^3^ In brief, non-silent mutations which were present in at least one sample with the following substitutions: C > A, C > G, C > T, T > A, T > C, T > G were used to analyze their distribution in the three groups of samples corresponding to NU/LGIN, HGIN, and UC. Fisher’s exact test was used to test the null hypothesis that they are equally distributed in the three groups of samples. The genomic context of SNVs including the two flanking bases on the 5’ and 3’ sides to each SNV was assembled and included 96 mutational patterns. The frequency of any fingerprints between groups of mucosal samples was tested by Wilcoxon Rank Sum tests. The Benjamini and Hochberg (BH) method was used to assess the false discovery rate (FDR).^29^ We used mutational fingerprints (V) in quadratic programming to estimate a weight score (H) for each mutational signature (W) from the Sanger Institute database (https://cancer.sanger.ac.uk/cosmic/signatures) as previously described.^3,4^ We used the matrix of canonical signatures (W) with the mutational profile of a sample (V) to compute the 30 by 1 vector (H) for each of the canonical signatures’ relative contributions to the sample mutagenesis profile by computing the following optimization:

minH (WH−V)T(WH−V) such that hi≥0 and Σi hi=1

Kruskal-Wallis test was used to test the null hypothesis of no difference in weight scores among the groups of samples. We applied bootstrapping to assess the contribution of mutational signatures in mucosal samples. Mutational fingerprints (V) for each sample were resampled with replacement and the weight scores were computed as above 2000 times. The one-sided empirical p value was computed as the percentage of weight scores that were equal or greater than the weight score in the resampling distribution.

### Phylogenetic Analysis and Modeling of Bladder Cancer Evolution

The phylogenetic tree was constructed by calculating the Hamming distances among the mucosal samples using a matrix of all nonsilent and silent mutations present in at least one sample by applying the maximum parsimony algorithm as previously described.^3^ In graphical representation of phylogenetic tree, each node corresponds to a population of cells and the length of the edge connecting the nodes is proportional to the number of mutations. A brunch represents a point in the evolution where two distinct population emerge. While the length of the branch is proportional to the number of mutations which are unique for each population.

To reconstruct the time of evolution from mucosal field effects to bladder cancer, the modified time-continuous Markov branching process with immigration and parsimonious principles was used as described previously with the following modifications.^10^ In brief, a mutation *j* appears at time $t_{0}^{j}$ in a progenitor cell of the urinary bladder urothelial lining and gives rise to a mutant clone. Mutant cells divide at rate λ_*j*_ (1/year), and after division, one cell enters self-renewal and the other differentiates with probability 1 – *s*_*j*_ or both cells enter self-renewal with probability *s*_*j*_. As a consequence, the mutant clone grows exponentially as exp (λ_*j*_*s*_*j*_*t*), where *t* is the age of the *j*-th mutant’s clone counted from $t_{0}^{j}$. The secondary clones expand, involving different areas of bladder mucosa at times $t_{i}^{j},i\geq 0$ modeled by a stochastic Poisson process with intensity *v* (1/yr).^30^ If the expected cell counts in the successive *j*-th mutant clones are denoted by $X_{i}^{j}(t)$, *i* = 0, 1, 2, …, and the number of haploid genomes in normal uroprogenitor cells are denoted by 2*N*, the corresponding VAFs $V_{i}^{j}(t)$ are defined as the ratios $V_{i}^{j}(t)=X_{i}^{j}(t)/(2N)$ and are computed as follows.^10^

$$E\left[V_{i}^{j}(t)\right]=\text{ exp }\left(\lambda_{j}s_{j}t\right){\left(\frac{\nu_{j}}{\nu_{j}+\lambda_{j}s_{j}}\right)}^{i}\int_{0}^{\left(\nu_{i}+\lambda_{j}s_{j}\right)t}\frac{u^{i-1}}{(i-1)!}\text{exp }(-u)du/(2N),i=0,1,2,\ldots$$

For any mutation *j* of age *t*_*j*_, the sequence of expectations $E\left[V_{i}^{j}\left(t_{j}\right)\right]$ *i* = 0, 1, 2, …, was computed to estimate the coefficients *a*_*j*_ = λ*s*_*j*_*t*_*j*_ and *b*_*j*_ = *v*_*j*_*t*_*j*_. With a cell division rate λ_*j*_ and migration rate *v*_*j*_, the parameter *b*_*j*_ is the proxy for mutation age *t*_*j*_, whereas the ratio *a*_*j*_/*b*_*j*_ is the proxy for selection coefficient *s*_*j*_. The coefficient *c* = 2*N* is a constant parameter representing an estimate of the number of uroprogenitor cells in the sampled area. The computations were performed for 10^2^ − 10^5^ uroprogenitor cells in the sampled mucosal area, which did not significantly change the time modelling results, but the best fit was obtained with 5 × 10^3^ uroprogenitor cells, for which the data are presented.

The objective is reconstruction of the evolution of mutational landscape from mucosal field effects to invasive cancer in the forward time by connecting the migration of urothelial cells to their proliferation rate. A parsimonious model assumes that the migration rate is proportional to the power *σ* of the proliferation rate, i.e. $\nu_{j}=\nu_{0}\rho_{j}^{\sigma }$. Hence the parameter *b*_*j*_ = *v*_*j*_*t*_*j*_, for mutant *j*, has the form $b_{j}=\nu_{0}\rho_{j}^{\sigma }t_{j}$, where *ρ*_*j*_ = λ_*j*_*s*_*j*_ (and *v*_0_ is a reference migration rate), has the form *a*_*j*_ = *t*_*j*_*ρ*_*j*_. Solving these two equations for *ρ*_*j*_ and *t*_*j*_ provides estimates for the proliferation rate (proxy for the selection coefficient) and mutation age of mutant *j*,

$$\rho_{j}={\left(b_{j}/\left(a_{j}\nu_{0}\right)\right)}^{1/(\sigma -1)},t_{j}=a_{j}^{1+1/(\sigma -1)}{\left(\nu_{0}/b_{j}\right)}^{1/(\sigma -1)}$$

We carried out a series of extensive parametric studies an found that estimates corresponding to high values of parameter *σ*, such as *σ* = 6, fit the chronology of different mutation classes, *α*, *β*, and γ, which is consistent with biological and clinical intuition. A fitting algorithm with the optimization programs and fminsearch and fminbnd in the MATLAB programming language was used to estimate the sequence of mutations in tumor development.^31–33^ The resulting time estimates were presented as bar diagrams representing the age of mutations and point charts for the corresponding selection coefficients.

### Mass Spectrometry-based Proteome Profiling

The global proteome profiling was processed as described previously with the following modifications.^34,35^ The frozen tissue was resuspended and lysed in 50mM Ammonium bicarbonate, 1 mM CaCl2 by 3 min of sonication, then 50 ug of tissue lysate were digested using 1 ug of trypsin for 12 hours at 37°C. Digested peptides concentration was measured using a colorimetric peptide assay kit (Thermo Fischer Scientific, cat. #23275) and 25 ug of peptides were separated in a home-made high pH reverse-phase C18 column in a pipet tip. Peptides were eluted and separated into fifteen fractions using a stepwise gradient of increasing acetonitrile (2, 4, 6, 8, 10, 12, 14, 16, 18, 20, 22, 24, 26, 28, 30% Acetonitrile) at pH 10 then combined to five fractions (2 + 12 + 12, 4 + 14 + 24, 6 + 16 + 26, 8 + 18 + 28, 10 + 20 + 30) and vacuum dried. The dried peptide samples were analyzed on Orbitrap Fusion mass spectrometers (Thermo Fisher Scientific) coupled with an Easy-nLC 1000 nanoflow LC system (Thermo Fisher Scientific). An in-housed trap column (2 cm × 100 μm i.d.) and a 5 cm × 150 μm capillary separation column packed with 1.9 μm Reprosil-Pur Basic C18 beads (Dr. Maisch, r119.b9.) was used for nano-HPLC separation in discontinuous gradient of 4–26% acetonitrile, 0.1% formic acid at a flow rate of 800 nl/min. The Mass spectrometry was operated in a data-dependent mode, acquiring fragmentation spectra of the top 30 strongest ions under the control of Xcalibur software version 4.1 (Thermo Fisher Scientific). The parental ion was acquired in the Orbitrap with a full MS range of 300–1400 m/z at the resolution of 120,000. Higher-energy collisional dissociation (HCD) fragmented MS/MS spectrum was acquired in ion-trap with rapid scan mode. The MS/MS spectra were searched against the target-decoy Human RefSeq database (release Jan. 21, 2020, containing 80,872 entries) in Proteome Discoverer 2.1 interface (Thermo Fisher) with Mascot algorithm (Mascot 2.4, Matrix Science). The precursor mass tolerance of 20 ppm and fragment mass tolerance of 0.5 Da was allowed. Two maximum missed cleavage, and dynamic modifications of acetylation of N-term and oxidation of methionine were allowed. Assigned peptides were filtered with a 1% false discovery rate (FDR) using Percolator validation based on q-value. The Peptide Spectrum Matches (PSMs) output from PD2.1 was used to group peptides onto gene level using ‘gpGrouper’ algorithm.^36^ An in-housed program, gpGrouper, uses a universal peptide grouping logic to accurately allocate and provide MS1 based quantification across multiple gene products. Gene-protein products (GPs) quantification was performed using the label-free, intensity-based absolute quantification (iBAQ) approach and then normalized to FOT (a fraction of the total protein iBAQ amount per experiment). FOT was defined as an individual protein’s iBAQ divided by the total iBAQ of all identified proteins within one experiment.

The missing values in the proteome recovery were replaced with half of the minimally detected value in the entire dataset. Following log2 transformation of this dataset, the differential analysis (t test) was performed comparing one specific group against all the remaining samples combined. This step was repeated for all the different experimental groups concerned.^37^ Any protein was deemed statistically altered expression it has a p-value of < 0.05 and greater than 1.5 fold change. The selected GPs were analyzed using Advaita Bio’s iPathwayGuide. A systems biology approach for pathway level analysis.^38^ Unsupervised hierarchical clustering of normalized protein expression values was performed in software R, and the results were visualized with R package “ComplexHeatmap”. Pearson’s correlation, mean centering, and average linkage were applied in all clustering applications.

### Targeted Metabolomics Analysis

The human bladder mucosal and tumor tissue lysates were used for metabolite extraction. The lysates from the urothelium harvested from normal human ureters from nephrectomies of patients with renal cancer without evidence of urothelial neoplasia were used as a reference. The mouse liver pool was used as Quality Control (QC), and was combined with 750 μL of internal standard (ISTD) mix. For the extraction of metabolites the geographically mapped cell suspensions and control reference samples were defrosted and processed by the liquid-liquid extraction method as described previously.^39–41^ In brief, after partitioning through ice-cold chloroform and water, organic and aqueous layers were carefully transferred into new glass vials. Proteins and lipids were removed from extracted samples using a 3K Amicon-Ultra filter (Millipore Corporation, Billerica, MA). The dried pellets were dissolved into methanol-water (50:50 v/v).

The extracted total metabolite samples were analyzed through high-throughput Liquid Chromatography-Mass Spectrometry (LC-MS/MS) techniques described previously.^39,40^ The chromatographic separation of extracted metabolites was performed through Hydrophilic Interaction Chromatography (HILIC) and Reverse Phase (RP) chromatography techniques. The metabolites were separated through the XBridge Amide HPLC column (3.5 μm, 4.6 × 100 mm, Waters, Milford, MA) in both ESI positive and negative mode. The details Lc methods were described in our earlier publications.^39,42,43^ The data was acquired via multiple reaction monitoring (MRM) using a 6495 Triple Quadrupole mass spectrometry coupled to an HPLC system (Agilent Technologies, Santa Clara, CA) through Agilent Mass Hunter Software.^39^ The acquired data were analyzed and integrated into each peak using Agilent Mass Hunter Quantitative Analysis software. The extracted peak area was log2 transformed and normalized by an isotopically labeled internal standard for each method.

There were in total 92 metabolites profiled and passing quality control. In addition to clustering and visualizing individual metabolites across samples by heatmaps, we calculated relative metabolic pathway activity level in each individual sample by single sample gene set enrichment analysis (ssGSEA). Specifically, we extracted 74 metabolic pathways from the Kyoto Encyclopedia of Genes and Genomes (KEGG) database. The ssGSEA method is an extension of GSEA method: it compares the difference in empirical cumulative distribution functions of the metabolites’ ranks inside and outside a given metabolic pathway to calculate an enrichment score for each sample. These ssGSEA enrichment scores could reflect how the metabolites in a pathway are coordinately upregulated or downregulated within a sample, with further normalization across all samples. We examined how the pathways were differentiated among the groups of NU/LGIN, HGIN, and UC using hierarchical clustering and heatmaps.

## Data Availability

The whole exome sequencing data both raw and analyzed were deposited on SRA: PRJNA1065919. The mass spectrometry data for proteome profiling have been deposited via the MASSIVE repository (MSV000085220) to the Proteome X change Consortium (http://proteomecentral.proteomexchange.org) with the dataset identifier PXD018341).

## References

[1] Sinjab A, Han G, Wang L, Kadara H (2020) field Carcinogenesis in Cancer Evolution: What the Cell Is Going On? Cancer Res 80:4888–4891. 10.1158/0008-5472.CAN-20-1956 33023945 PMC7669616

[2] Curtius K, Wright NA, Graham TA (2018) An evolutionary perspective on field cancerization. Nat Rev Cancer 18:19–32. 10.1038/nrc.2017.102 29217838

[3] Bondaruk J, Jaksik R, Wang Z, Cogdell D, Lee S, Chen Y, Dinh KN, Majewski T, Zhang L, Cao S (2022) The origin of bladder cancer from mucosal field effects. iScience 25, 104551. 10.1016/j.isci.2022.104551 35747385 PMC9209726

[4] Majewski T, Yao H, Bondaruk J, Chung W, Lee S, Lee JG, Zhang S, Cogdell D, Yang G, Choi W (2019) Whole-Organ Genomic Characterization of Mucosal field Effects Initiating Bladder Carcinogenesis. Cell Rep 26:2241–2256e2244. 10.1016/j.celrep.2019.01.095 30784602

[5] Lawson ARJ, Abascal F, Coorens THH, Hooks Y, O’Neill L, Latimer C, Raine K, Sanders MA, Warren AY, Mahbubani KTA (2020) Extensive heterogeneity in somatic mutation and selection in the human bladder. Science 370:75–82. 10.1126/science.aba8347 33004514

[6] Strandgaard T, Lamy NI, Christensen P, Borg E, Thomsen H, Thomsen MBH, Jensen M, Bjerggaard Jensen JB, Dyrskjøt J, Dyrskjot L L (2020) Mutational analysis of field cancerization in bladder cancer. Bladder Cancer 6:253264

[7] Thomsen MBH, Nordentoft I, Lamy P, Vang S, Reinert L, Mapendano CK, Hoyer S, Orntoft TF, Jensen JB, Dyrskjot L (2017) Comprehensive multiregional analysis of molecular heterogeneity in bladder cancer. Sci Rep 7:11702. 10.1038/s41598-017-11291-0 28916750 PMC5600970

[8] Strandgaard T, Nordentoft I, Birkenkamp-Demtroder K, Salminen L, Prip F, Rasmussen J, Andreasen TG, Lindskrog SV, Christensen E, Lamy P (2024) field Cancerization Is Associated with Tumor Development, T-cell Exhaustion, and Clinical Outcomes in Bladder Cancer. Eur Urol 85:82–92. 10.1016/j.eururo.2023.07.014 37718188

[9] Dinh KN, Kimmel JR, Lambert M, Tavare A S (2020) Statistical Inference for the Evolutionary History of Cancer Genomes. Stat Sci 35:129–144 10.1214/19-sts7561 PMC12519734 41098675

[10] Lange K (2010) Branching Processes. In Applied Probability, (Springer), pp. 217–245

[11] Davalieva K, Kiprijanovska S, Ivanovski O, Trifunovski A, Saidi S, Dimovski A, Popov Z (2023) Proteomics Profiling of Bladder Cancer Tissues from Early to Advanced Stages Reveals NNMT and GALK1 as Biomarkers for Early Detection and Prognosis of BCa. Int J Mol Sci 24. 10.3390/ijms241914938 PMC10573217 37834386

[12] Yao Z, Xu N, Shang G, Wang H, Tao H, Wang Y, Qin Z, Tan S, Feng J, Zhu J (2023) Proteogenomics of different urothelial bladder cancer stages reveals distinct molecular features for papillary cancer and carcinoma in situ. Nat Commun 14. 10.1038/s41467-023-41139-3 PMC10499981 37704624

[13] Tabaei S, Haghshenas MR, Ariafar A, Gilany K, Stensballe A, Farjadian S, Ghaderi A (2023) Comparative proteomics analysis in different stages of urothelial bladder cancer for identification of potential biomarkers: highlighted role for antioxidant activity. Clin Proteom 20. 10.1186/s12014-023-09419-8 PMC10373361 37501157

[14] Alexandrov LB, Nik-Zainal S, Wedge DC, Aparicio SA, Behjati S, Biankin AV, Bignell GR, Bolli N, Borg A, Borresen-Dale AL (2013) Signatures of mutational processes in human cancer. Nature 500:415–421. 10.1038/nature12477 23945592 PMC3776390

[15] Inamoto T, Shah JB, Kamat AM (2009) Friend or foe? Role of peroxisome proliferator-activated receptor-gamma in human bladder cancer. Urol Oncol 27:585–591. 10.1016/j.urolonc.2008.11.002 19162510

[16] Li Y, Pan Y, Zhao X, Wu S, Li F, Wang Y, Liu B, Zhang Y, Gao X, Wang Y, Zhou H (2024) Peroxisome proliferator-activated receptors: A key link between lipid metabolism and cancer progression. Clin Nutr 43:332–345. 10.1016/j.clnu.2023.12.005 38142478

[17] Damaghi M, West J, Robertson-Tessi M, Xu L, Ferrall-Fairbanks MC, Stewart PA, Persi E, Fridley BL, Altrock PM, Gatenby RA (2021) The harsh microenvironment in early breast cancer selects for a Warburg phenotype. Proc Natl Acad Sci U S A 118. 10.1073/pnas.2011342118 PMC7826394 33452133

[18] Gnocchi D, Nikolic D, Paparella RR, Sabba C, Mazzocca A (2023) Cellular Adaptation Takes Advantage of Atavistic Regression Programs during Carcinogenesis. Cancers (Basel) 15. 10.3390/cancers15153942 PMC10416974 37568758

[19] Gnocchi D, Sabba C, Mazzocca A (2023) Lactic acid fermentation: A maladaptive mechanism and an evolutionary throwback boosting cancer drug resistance. Biochimie 208:180–185. 10.1016/j.biochi.2023.01.005 36638953

[20] Jia D, Lu M, Jung KH, Park JH, Yu L, Onuchic JN, Kaipparettu BA, Levine H (2019) Elucidating cancer metabolic plasticity by coupling gene regulation with metabolic pathways. Proc Natl Acad Sci U S A 116:3909–3918. 10.1073/pnas.1816391116 30733294 PMC6397570

[21] Amara CS, Vantaku V, Lotan Y, Putluri N (2019) Recent advances in the metabolomic study of bladder cancer. Expert Rev Proteom 16:315–324. 10.1080/14789450.2019.1583105 PMC6538267 30773067

[22] Kami Reddy KR, Piyarathna DWB, Kamal AHM, Putluri V, Ravi SS, Bollag RJ, Terris MK, Lotan Y, Putluri N (2022) Lipidomic Profiling Identifies a Novel Lipid Signature Associated with Ethnicity-Specific Disparity of Bladder Cancer. Metabolites 12. 10.3390/metabo12060544 PMC9230655 35736477

[23] Piyarathna DWB, Rajendiran TM, Putluri V, Vantaku V, Soni T, von Rundstedt FC, Donepudi SR, Jin F, Maity S, Ambati CR (2018) Distinct Lipidomic Landscapes Associated with Clinical Stages of Urothelial Cancer of the Bladder. Eur Urol Focus 4:907–915. 10.1016/j.euf.2017.04.005 28753886 PMC5650548

[24] Sen S, Zhou H, Zhang RD, Yoon DS, Vakar-Lopez F, Ito S, Jiang F, Johnston D, Grossman HB, Ruifrok AC (2002) Amplification/overexpression of a mitotic kinase gene in human bladder cancer. J Natl Cancer Inst 94:1320–1329. 10.1093/jnci/94.17.1320 12208897

[25] Lee S, Jeong J, Majewski T, Scherer SE, Kim MS, Tuziak T, Tang KS, Baggerly K, Grossman HB, Zhou JH (2007) Forerunner genes contiguous to RB1 contribute to the development of in situ neoplasia. Proc Natl Acad Sci U S A 104:13732–13737. 10.1073/pnas.0701771104 17702869 PMC1949496

[26] Cancer IAfRo, Moch H, Reuter five (2016) WHO Classification of Tumours of the Urinary System and Male Genital Organs. International Agency for Research on Cancer

[27] Brierley JD, Gospodarowicz MK, Wittekind C (2016) TNM Classification of Malignant Tumours, 8th Edition (Wiley-Blackwell)

[28] Richter J, Jiang F, Gorog JP, Sartorius G, Egenter C, Gasser TC, Moch H, Mihatsch MJ, Sauter G (1997) Marked genetic differences between stage pTa and stage pT1 papillary bladder cancer detected by comparative genomic hybridization. Cancer Res 57:2860–2864 9230190

[29] Benjamini Y, Hochberg Y (1995) Controlling the False Discovery Rate - a Practical and Powerful Approach to Multiple Testing. J R Stat Soc B 57:289–300. 10.1111/j.2517-6161.1995.tb02031.x

[30] Last G, Penrose M (2017) Lectures on the Poisson Process. Cambridge University Press

[31] Forsythe GE, Malcolm MA, Moler CB (1977) Computer methods for mathematical computations. Prentice-Hall)

[32] Lagarias JC, Reeds JA, Wright MH, Wright PE (1998) Convergence properties of the Nelder-Mead simplex method in low dimensions. Siam J Optimiz 9:112–147. 10.1137/S1052623496303470

[33] Brent RP (1972) Algorithms for minimization without derivatives. Prentice-Hall)

[34] Jung SY, Choi JM, Rousseaux MW, Malovannaya A, Kim JJ, Kutzera J, Wang Y, Huang Y, Zhu W, Maity S (2017) An Anatomically Resolved Mouse Brain Proteome Reveals Parkinson Disease-relevant Pathways. Mol Cell Proteom 16:581–593. 10.1074/mcp.M116.061440 PMC5383780 28153913

[35] Yu Y, Gao SM, Guan Y, Hu PW, Zhang Q, Liu J, Jing B, Zhao Q, Sabatini DM, Abu-Remaileh M (2024) Organelle proteomic profiling reveals lysosomal heterogeneity in association with longevity. Elife 13. 10.7554/eLife.85214 PMC10876212 38240316

[36] Saltzman AB, Leng M, Bhatt B, Singh P, Chan DW, Dobrolecki L, Chandrasekaran H, Choi JM, Jain A, Jung SY (2018) gpGrouper: A Peptide Grouping Algorithm for Gene-Centric Inference and Quantitation of Bottom-Up Proteomics Data. Mol Cell Proteom 17:2270–2283. 10.1074/mcp.TIR118.000850 PMC6210220 30093420

[37] Mindikoglu AL, Abdulsada MM, Jain A, Choi JM, Jalal PK, Devaraj S, Mezzari MP, Petrosino JF, Opekun AR, Jung SY (2020) Intermittent fasting from dawn to sunset for 30 consecutive days is associated with anticancer proteomic signature and upregulates key regulatory proteins of glucose and lipid metabolism, circadian clock, DNA repair, cytoskeleton remodeling, immune system and cognitive function in healthy subjects. J Proteom 217:103645. 10.1016/j.jprot.2020.103645 PMC7429999 31927066

[38] Draghici S, Khatri P, Tarca AL, Amin K, Done A, Voichita C, Georgescu C, Romero R (2007) A systems biology approach for pathway level analysis. Genome Res 17:1537–1545. 10.1101/gr.6202607 17785539 PMC1987343

[39] Gohlke JH, Lloyd SM, Basu S, Putluri V, Vareed SK, Rasaily U, Piyarathna DWB, Fuentes H, Rajendiran TM, Dorsey TH (2019) Methionine-Homocysteine Pathway in African-American Prostate Cancer. JNCI Cancer Spectr 3:pkz019. 10.1093/jncics/pkz019 PMC6489686 31360899

[40] Putluri N, Shojaie A, Vasu VT, Vareed SK, Nalluri S, Putluri V, Thangjam GS, Panzitt K, Tallman CT, Butler C (2011) Metabolomic profiling reveals potential markers and bioprocesses altered in bladder cancer progression. Cancer Res 71:7376–7386. 10.1158/0008-5472.CAN-11-1154 21990318 PMC3249241

[41] Vantaku V, Dong J, Ambati CR, Perera D, Donepudi SR, Amara CS, Putluri V, Ravi SS, Robertson MJ, Piyarathna DWB (2019) Multi-omics Integration Analysis Robustly Predicts High-Grade Patient Survival and Identifies CPT1B Effect on Fatty Acid Metabolism in Bladder Cancer. Clin Cancer Res 25:3689–3701. 10.1158/1078-0432.CCR-18-1515 30846479 PMC6571061

[42] Chakraborty S, Lulla A, Cheng X, Yeo JY, Mandal J, Yang T, Mei X, Saha P, Golonka RM, Yeoh BS (2023) Conjugated bile acids are nutritionally re-programmable antihypertensive metabolites. J Hypertens 41:979–994. 10.1097/HJH.0000000000003423 37071431 PMC10158603

[43] Vantaku V, Putluri V, Bader DA, Maity S, Ma J, Arnold JM, Rajapakshe K, Donepudi SR, von Rundstedt FC, Devarakonda V (2020) Epigenetic loss of AOX1 expression via EZH2 leads to metabolic deregulations and promotes bladder cancer progression. Oncogene 39:6265–6285. 10.1038/s41388-019-0902-7 31383940 PMC8058741

